# Continuous Gum

**Published:** 1856-01

**Authors:** 


CONTINUOUS GUM.

The *Dental Obturator* for August, 1855, contains a well written article on this subject, giving somewhat in detail the advantage secured by this kind of work, and after answering some of the special objections often urged, the author of the article, Dr. J. S. Clark, notices the popular objections in the following manner:

One says, "I tried it and do not like it, for the gum does not come out all alike. It will spot, and besides it checks and cracks." Clean fingers and articles used in manipulation will remedy the first, and proper care in heating up and cooling off will remedy the latter. Another says, "it breaks and scales off in the mouth." Brace it, as instructed, and that will not occur. But after all, these objections amount to very little, for they are the objections arising from the attempts of beginners, or the mistakes of the unskillful. We have no doubt that there are many who find no difficulty in gold work, who yet find a great deal in attempting this, and most sincerely do we say, that we believe there are many who do passable work in gold, that never can produce a good piece with continuous gum. Also, that there are many other objectors who having found out the difficulties, and perhaps suffered a few failures, find it more convenient to stick to the old style in all cases, because it is the easiest.

We regard the introduction of this improvement, (for we shall call it such,) as of great benefit thus far, and promising more for the future to the profession. It has improved the manufacture of teeth, both in shape and composition, and thus we are benefitted.

But above all, it has done much to artistise, (so to speak,) our productions. We want all for our profession that tends to draw the line between dentistry as a science and art, the result of genius and knowledge joined to high motives of livelong benevolence, and the misnomer of dentistry as a "trade," a bartering with human credulity, a stepping stone to a better speculation. When will the keen sensibilities of our natures cease to feel the pain of insult presented (unconsciously) in our consultations, arising from false estimates of our profession, produced by unfortunate contact with what was called dentistry, but which proved to be malpractice ?

Never, never, till she can stand forth the undeviating dispensor of good works, the true friend to the greatest good to suffering humanity, by an exactitude of adaption that knows no short road pointed out by convenience, or a miserable truckling to false and ignorant tastes. Who is responsible for these false estimates ? Where is that pure stream to issue from, that shall wash away our plague spots ?

Just as surely as will the pure mountain rill start on its cheering mission, will demonstrated truth of action and deed send forth a healing stream, that ere long will well up into our laboratories and our operating rooms, cheering us in our daily toil.

We are not bound, nor do we advocate the attempt to teach our patients dentistry, but we are responsible to the profession in a great measure, for the main influence that is wielded by our patients in regard to us as a profession. The dentist of proper qualifications and adaptations to the practice never yet commenced with faithfulness, and persevered by assiduous attention to the best good of his patients that the great influence wielded for our profession by his patients, was not for good, and by whose labors the profession was not enriched in character and usefulness.

